# Development of the Pipette-Tip Micro-Solid-Phase Extraction for Extraction of Rutin From *Moringa oleifera* Lam. Using Activated Hollow Carbon Nanospheres as Sorbents

**DOI:** 10.1155/2024/2681595

**Published:** 2024-09-27

**Authors:** Dakalo Lorraine Ndou, Bonakele Patricia Mtolo, Adivhaho Khwathisi, Ashwell Rungano Ndhlala, Nikita Tawanda Tavengwa, Ntakadzeni Edwin Madala

**Affiliations:** ^1^ Department of Chemistry Faculty of Science Engineering and Agriculture University of Venda, Private Bag X5050, Thohoyandou 0950, South Africa; ^2^ DSI-NRF Centre of Excellence in Strong Materials School of Chemistry University of the Witwatersrand, Johannesburg 2050, South Africa; ^3^ Department of Biochemistry Faculty of Science Engineering and Agriculture University of Venda, Private Bag X5050, Thohoyandou 0950, South Africa; ^4^ Green Biotechnologies Research Centre Department of Plant Production Soil Science and Agricultural Engineering University of Limpopo, Private Bag X1106, Sovenga 0727, South Africa

**Keywords:** hollow carbon nanospheres, *Moringa oleifera*, pipette-tip micro-solid-phase extraction (PT-*µ*SPE), rutin, sorbents

## Abstract

Herein, a micro-solid-phase extraction (*μ*SPE) method was developed using a pipette tip for rutin extraction, employing activated hollow carbon nanospheres (HCNSs) as the sorbent. Characterization of the activated carbon nanospheres through TGA, FTIR, and SEM analysis confirmed the success of the activation process. The study demonstrated the efficacy of PT-*μ*SPE in rutin extraction under pH 2 conditions with a standard concentration of 2 mg·L^−1^. The optimal mass of HCNSs was found to be 2 mg, and a loading volume of 500 *μ*L resulted in the maximum recovery of rutin. Propan-2-ol was the best elution solvent with 15 aspirating/dispensing cycles. The correlation of determination (*R*^2^) for the calibration curve was found to be 0.9991, and the LOD and LOQ values were 0.604 and 1.830 mg·L^−1^, respectively. The applicability of the method was demonstrated by extracting rutin from a complex *Moringa oleifera* leaf extract with the relative standard deviation (RSD) of 3.26%, thereby validating this method as feasible for the extraction of useful bioactive compounds from complex plant samples.

## 1. Introduction

Conventional methods of extraction of analytes from different matrices have proven to make use of large amounts of reagents and solvents which generate large amount of waste and have long analysis times, resulting in undesirable consequences to both humans and their environment [1–3]. These drawbacks have resulted in the need for the development of green sample preparation techniques to replace the conventional methods with the simpler and environmentally friendly techniques [4–6]. Environmentally friendly extraction methods such as microwave-assisted extraction (MAE) [7], ultrasound-assisted extraction (UAE) [8], supercritical fluid extraction (SFE) [9], and pressurized liquid extraction (PLE) [10] have been used for the extraction of bioactive compounds. The extraction methods facilitated by these advancements eliminate or minimize the use of harmful solvents and reduce extraction time. They improve the efficiency of extraction, resulting in higher yields and enhanced extract quality [3, 11].

Microextraction techniques make use of a small volume of the extracting solvents relative to the volume of the samples, thus implying high enrichment factors [12, 13]. Miniaturized techniques are of advantage because they provide a straightforward procedure, faster analysis, higher extraction performance, and reduced amount of sample required [14, 15]. These techniques also use recent advances in the synthesis of new sorbent materials and the use of greener extraction solvents [14, 16]. Microextraction techniques can be divided into three broad groups depending on the type of the extracting phase, i.e., solid-phase microextraction (SPME) [17], liquid-phase microextraction (LPME) [18], and membrane microextraction [19]. SPME is a modern and nonexhaustive sample preparation technique which integrates sampling, preconcentration, and extraction in a single step, and the analytes can be directly introduced to analytical instruments like chromatographic systems [17, 20].

Pipette-tip micro-solid-phase extraction (PT-*µ*SPE) represents a novel form of miniaturized SPE. It serves as a straightforward, portable, and swift sample pretreatment method applicable to proteins, peptides, and drugs [21–23]. In this technique, a microscale amount of a sorbent is packed inside a pipette tip with the aim of reducing volume of solvents and samples [21, 24, 25]. The extraction process begins by drawing the sample solution into the tip and then dispensing it back into the sample tube. By replicating the aspirating/dispensing cycles, the extraction procedure reaches equilibrium, and this makes the procedure controllable, stable, and smooth [21, 26–28]. An appropriate solvent is then used to desorb the analytes. To achieve quantitative desorption, this step may be repeated several times [26]. It has been used for the determination and extraction of analytes such as pharmaceuticals, bioanalytical molecules, proteins, and metals in various matrices [29]. Sorbents such as multiwalled carbon nanotubes (MWCNTs), silica, molecularly imprinted polymers (MIPs), graphene oxide (GO), and carbonaceous materials have been used as sorbents [30–32]. Carbon nanotubes have been used as SPE sorbents for the extraction of drugs [33], pesticides, or natural compounds in different media such as biological fluids [34], environment [35], and plants or animal organs [36, 37]. Carbonaceous materials such as graphene, carbon nanotubes, and porous carbons have been thoroughly investigated. These materials are advantageous because of their low density, high porosity, and large surface area, and they have thus attracted many scientific and technological interests. They have thus been applied in various areas such as catalyst supports, adsorption, separation systems, and nanoreactors [38].

In this study, PT-*µ*SPE was used as the extraction method for extracting a highly bioavailable flavonoid, rutin, from a multipurpose *Moringa oleifera* plant. Activated hollow carbon nanospheres (HCNSs) were used as sorbent material. This is the first study to report on the extraction of rutin from *M. oleifera* using PT-*µ*SPE.

## 2. Materials and Methods

### 2.1. Chemicals and Reagents

Rutin (anhydrous ≥ 99% purity) was purchased from Sigma-Aldrich (Modderfontein, South Africa), sodium hydroxide pellets and hydrochloric acid (32%) were purchased from Merck (Modderfontein, South Africa), and potassium chloride and propan-2-ol were purchased from Associated Chemical Enterprises (Johannesburg, South Africa). Ultrapure water (0.005 *μ*S, 18 mΩ) was used for the preparation of the standard solutions. Methanol and acetonitrile were purchased from Fluka (Steinheim, Germany).

A Thermo Orion Star A121 Portable pH meter was used for pH measurements, and a DIAB MX-RL-Pro dragon shaker was also used.

### 2.2. Instruments for Characterization

FTIR spectroscopy analysis was done using a Bruker Tensor 27 Fourier Transform Infrared Spectrometer. The absorption measurements of electromagnetic radiation were between 400 and 4000 cm^−1^. The surface morphology of the samples was performed using SEM on a ZEISS SIGMA 03-39 FESEM operating at 20 kV. A small amount of the sample was spread on carbon tape and coated with a layer of carbon and gold/palladium alloy before analysis. Thermal analysis was done using Perkin Elmer TGA 6000 thermogravimetric analyzer. A mass of approximately ± 9 mg inside a ceramic pan was placed inside the instrument furnace and heated in the temperature range of 35°C to 900°C at a rate of 10°C min^−1^ under a flow of air (20 mL·min^−1^) gas while monitoring the change in mass.

### 2.3. Preparation of Stock and Real Solutions

A working stock solution (5 mg°L^−1^) was prepared from a standard stock solution of 500 mg°L^−1^ and made to the mark using methanol. Working standard solutions of 0.5, 1, 1.5, and 2 mg°L^−1^ were prepared from the stock solution and made to the mark using the same diluent. A series of calibration standard solutions (0.7–50 mg°L^−1^) were prepared by appropriate dilutions of the standard stock solution. Extractions from *M. oleifera* were performed using 80% MeOH. The supernatant solution was stored in a refrigerator at 4°C when not in use.

### 2.4. Synthesis of Polystyrene Spheres

The polystyrene spheres (PSs) were prepared by adding 0.2 g polyvinylpyrrolidone (PVP), 50 mL water, and 200 mL ethanol in a 500-mL round-bottom flask. After all the PVP had dissolved, styrene (16 mL) was added to the mixture and stirred for 15 min. A solution of potassium persulfate (0.3 g) dissolved in distilled water (20 mL) was added to the flask while stirring. The mixture was then refluxed at 80°C while stirring for 24 h. The final product was filtered by centrifuge at 18,000 rpm for 15 min and washed repeatedly with ethanol. The resulting product was dried at 60°C for 4 h, crushed into a fine powder, and denoted as PSs.

### 2.5. Synthesis of HCNSs

The HCNSs were synthesized by coating the PSs template with resorcinol-formaldehyde (RF) polymer using the following procedure: PSs (2 g) were dispersed in a mixture of ethanol (100 mL), ammonia solution (25%, 3 mL), and distilled water (50 mL) in a 500 mL bottle by ultrasonication. A solution containing formaldehyde (37%, 3 mL), resorcinol (1 g), cetyltrimethylammonium bromide (CTAB) (1.5 g), and ethanol (75 mL) was added slowly to the latter, and the resulting solution was stirred for 12 h at room temperature. This was followed by hydrothermal treatment in an oil bath at 80°C for 24 h. A layer of RF polymer was formed around the PSs. The resulting product was filtered and washed several times with ethanol/water and dried in the oven at 60°C for 12 h. The sample was then crushed into a fine powder and denoted as PSs@RF. The dry composite (PSs@RF) was then heated inside a horizontal chemical vapor deposition (CVD) furnace in a one-step process to remove the template and carbonize the material. This was achieved by heating the composites under a controlled nitrogen flow (80 mL·min^−1^) at a heating rate of 5°C·min^−1^ to 350°C and kept isothermal for 1 h to decompose the PSs followed by carbonization of the RF polymer to form HCNSs at 600°C for 2 h. The resulting product was denoted as HCNSs.

### 2.6. Activation of HCNSs With NaOH

A modified method by Song et al. [39] was used. To activate the HCNSs for extraction of rutin, 120 mg of HCNSs powder was dispersed in NaOH (0.5 M, 100 mL) solution. The mixture was stirred on a magnetic stirrer for 1 h at room temperature. The product was filtered and washed with a large amount of deionized water to remove residual NaOH until the pH of the filtrate was near neutral. The activated HCNSs samples were dried in an oven at 60°C for 12 h.

### 2.7. Miniaturized Pipette-Tip Preparation

A modified method by Tavengwa et al. [40] was used. A fixed mass of glass wool (5 mg) was preloaded into each polypropylene pipette tip (1000 *μ*L). The activated HCNSs (≈2 mg) were added into the preloaded polypropylene pipette tip. An aliquot rutin standard solution (200 *μ*L) dissolved in methanol was aspirated onto the HCNSs and dispensed back into the same sample tube.

#### 2.7.1. Optimization of the Extraction Conditions

Parameters capable of influencing the recovery of rutin from the standard methanol solutions were investigated using HCNSs PT-*µ*SPE. The effect of sample pH (adjusted using 0.1 M HCl and 0.1 M NaOH), number of loading cycles, concentration of sample, sample loading volume, mass of sorbent, and choice of eluent were investigated and optimized by using the one variable at a time (OVAT) approach. All the experiments were done in triplicate.

### 2.8. Point of Zero Charge (pHpzc)

For the determination of pHpzc, a 0.01 M KCl solution was prepared, and its initial pH (pH_i_) was adjusted to between 2 and 11 by the addition of 0.1 M HCl and 0.1 M NaOH in different test tubes. A volume of 10 mL of the pH-adjusted KCl solution and 2 mg of HCNSs and HCNS-OH were mixed at room temperature for 25 h. After the elapsed time, the solution was separated from the HCNSs and HCNS-OH by centrifugation. After decanting, the final pH (pH_f_) of the solution was measured.

### 2.9. HPLC Chromatographic Method Conditions

An HPLC-PDA (LC-2030C, Shimadzu Corporation, Kyoto, Japan) fitted with a Shim-pack GIST column (150 mm × 4.6 mm, particle size 5.0 *μ*m) was used for the analysis. The column oven was set at a constant temperature of 50°C. A 20 *μ*L injection volume was employed, and the analyte was subjected to over a 16-min binary gradient at a flow rate of 0.8 mL°min^−1^. The mobile phase was a binary solvent mixture consisting of 0.1% formic acid in water (Eluent A) and 0.1% formic acid in methanol (Eluent B). The concentration of solvent B was gradually increased between 3 and 16 min to determine elution rutin in the samples. Briefly, Eluent B was held at 10% from 0 to 3 min, thereafter increased gradually from 10% to 60% between 3 and 6 min, and further elevated to 90% between 6 and 7 min. Eluent B was then maintained at 90% between 7 and 10 min. The concentration of B was returned to 10% in 4 min, and the column was allowed to reequilibrate at 10% until 16 min, in preparation for the next run. Chromatography elution was monitored using a photodiode array (PDA) detector operating at 190–700 nm, and rutin was specifically monitored at its maximum absorption peak at 359 nm.

## 3. Results and Discussion

### 3.1. Characterization of the Sorbent Materials

#### 3.1.1. Thermostability Analysis

**Figure 1:**
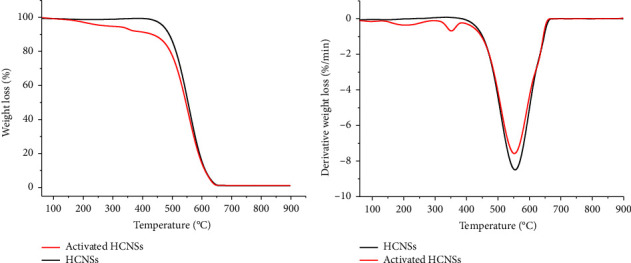
The TGA (a) and DTG (b) profiles of the raw and activated HCNSs.

The thermal stability of the raw HCNSs and the activated HCNSs was analyzed using thermogravimetric analysis (TGA).  Figure 1 displays the TGA/DTG profiles of the HCNSs samples. The continuous weight loss denotes the breaking of the chemical bonds on the surface of the HCNSs with an increase in temperature. The onset decomposition temperature of the raw HCNSs occurred at *ca.* 443°C, whereas the activated HCNSs began to decompose at *ca.* 423°C (Figure 1(a)). This implied that the raw HCNSs were more thermally stable than the activated HCNSs. The lower thermal stability of the activated HCNSs can be associated with the defects in the carbon framework induced by NaOH treatment. The peak observed at *ca.* 208°C in the derivative profile of the activated HCNSs can be related to the loss of moisture (Figure 1(b)). A similar observation was reported by Cervantes-Uc et al. [41] in a polymethacrylate polymer containing carboxylic groups, wherein the dehydration of the carboxylic acid functional groups was reported to be at 250°C. Another study by Mohamed et al. [42] observed a decomposition at 250°C in the TGA thermogram of nitrogen-doped microporous carbons from two monomers that contained carboxylic groups and associated it with the partial decarboxylation of the COOH groups. The second peak observed at ca. 353°C in the DTG profile of the activated HCNSs could be attributed to the removal of the oxygen-containing functional groups. The raw and activated HCNSs exhibited a similar maximum weight loss at 550°C and 554°C, respectively, associated with the decomposition of the carbon material. Moreover, complete decomposition of the carbon core was observed at temperatures > 650°C with no residues noted at 900°C, which signified the absence of impurities in the samples. Mtolo [43] also observed a complete decomposition of the carbon material with no residues and attributed it to be due to the purity of the material. A similar observation was observed by Yan et al. [44] wherein a strong weight loss of the HCNSs was observed at a temperature range of 200°C to 700°C. On the other hand, Tavengwa et al. [40] observed a similar trend, wherein the oxidized carbon nanofibers (CNFs) were less stable than the raw CNFs. This observation was attributed to the disorder caused by the presence of the hydroxyl functional groups.

#### 3.1.2. Functional Group Analysis

**Figure 2:**
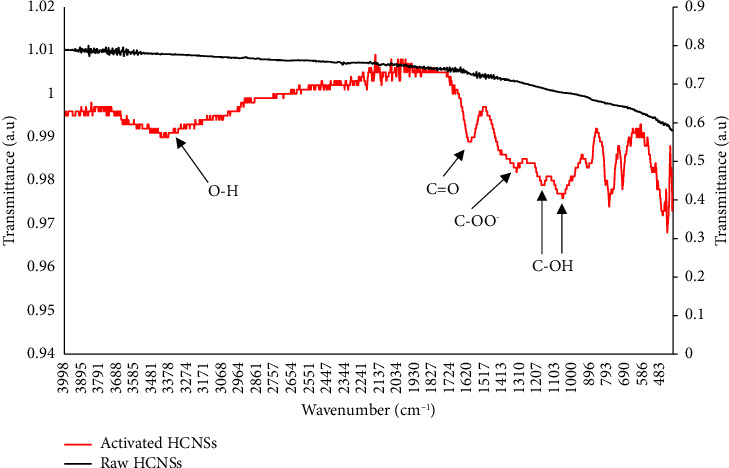
FTIR spectra of the raw HCNSs and the activated HCNSs.

The FTIR spectra of the raw HCNSs and the activated HCNSs are shown in Figure 2. The surface of the raw HCNSs was observed to not contain any functional groups as it is a pure carbon material. Upon activation of the HCNSs, new functional groups were observed in the spectrum as seen in Figure 2. The FTIR spectrum for the activated HCNSs shows a C=O stretching vibration of the carbonyl group at 1610 cm^−1^; a stretching vibration of a deprotonated carboxyl group, -COO^−^, at 1324 cm^−1^; OH bending vibrations in C-OH at 1164 cm^−1^; and a C-OH stretching at 1052 cm^−1^. A broad O-H stretching vibration in the range 3000–3700 cm^−1^, which is due to the enrichment of the hydroxyl groups on the surface of HCNSs after NaOH activation, is also observed. In the current study, these observations confirmed that the surface of the HCNSs was successfully activated by NaOH.

#### 3.1.3. Surface Morphological Studies

**Figure 3:**
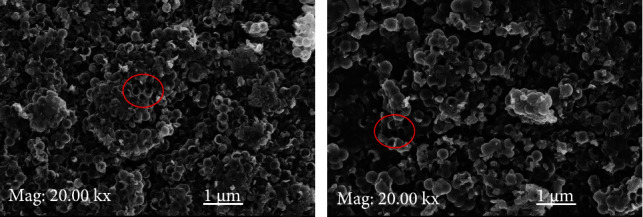
SEM images of (a) raw HCNSs and (b) activated HCNSs.

SEM images (Figure 3(a)) show the raw HCNSs which are spherical in shape and are also hollow (as indicated in the image). The spheres are grouped together due to the reactive bonds on their surface. This observation is similar to what was observed by Nieto-Marquez et al. [45]. In their study, they observed that HCNSs appeared as a conglomeration of spherical bodies, and this was attributed to the presence of the reactive bonds on their surface which provides them with a high surface reactivity. Boufades et al. [46] also attributed the conglomeration of the HCNSs to their susceptibility to temperature changes. According to Nieto-Marquez et al. [45], the graphite sheets that form the HCNSs are unclosed shells that have waving flakes that follow the curvature of the sphere, thus creating open edges at the surface. The graphitic flakes are reported to provide reactive “dangling bonds,” which are proposed to enhance the surface reactivity of the spheres.  Figure 3(b) shows the SEM image of the activated HCNSs. The activation of the HCNSs using NaOH increases the presence of the reactive bonds on the surface of the spheres, thereby providing them with a higher surface reactivity. This, therefore, means that the spherical bodies will coalesce to form bigger conglomerate because of the functional groups that can result in hydrogen bonding. This is what was observed in this study which confirmed the activation of the surface of the HCNSs.

### 3.2. Effect of Aspirating/Dispensing Cycles

**Figure 4:**
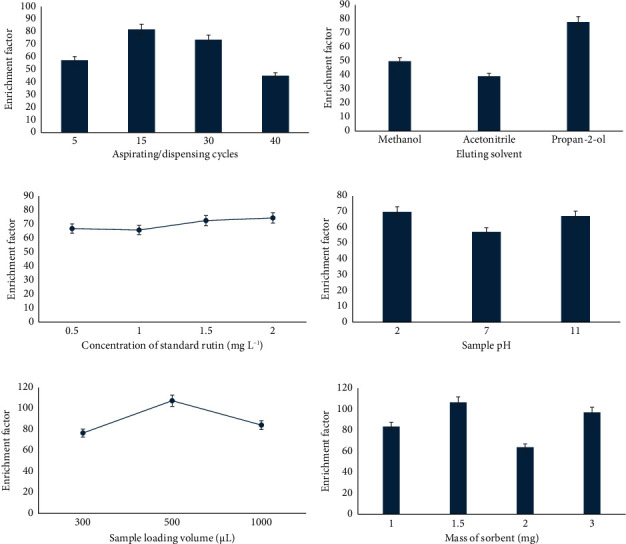
Effect of (a) aspirating/dispensing cycles, (b) eluting solvent, (c) concentration of standard rutin, (d) sample pH, (e) sample loading volume, and (f) mass of sorbent (*n* = 3, SD).

The enrichment of rutin was investigated by optimizing the loading cycles from 5 to 40. The number of aspirating/dispensing cycles was found to be critical for the enrichment of rutin using PT-*µ*SPE, and the maximum amount of rutin extracted was observed after 15 aspirating/dispensing cycles as observed in Figure 4(a) (extraction conditions: initial concentration of rutin = 0.5 mg·L^−1^, sample loading volume = 1000 *μ*L, elution solvent = MeOH, sample pH = 6, and mass of sorbent = 2 mg). Increasing the number of cycles to above 15 resulted in a decrease in the amount of rutin enriched on the sorbent bed. This observation could be because as the number of cycles increase, rutin is desorbed from the sorbent bed and eluted with the loading solvent due to the sheer stress exerted with increasing pipetting cycles. Therefore, throughout the experiment, 15 aspirating/dispensing cycles were used. A reduction in the extraction of rutin that was observed after 15 aspirating/dispensing cycles could be probably due to the elution of the analyte during the dispensing action which was undesirable on the loading stage. A similar trend was observed by Tavengwa et al. [47]. Abbaszadehbezi et al. [48] concluded that at a high number of aspirating/dispensing cycles, the back extraction of analytes from the sorbent bed to the sample solution might occur, which causes a decrease in the recovery of analyte.

### 3.3. Effect of Eluting Solvent

The elution solvent was also found to have a significant role in the enrichment of rutin in PT-*µ*SPE (Figure 4(b)). The choice of the eluting solvent should be able to elute the most amount of rutin from the pipette tip and is hence available for analysis. Herein, three polar organic solvents (listed in order of increasing polarity) were investigated: *i*-PrOH (3.92), MeOH (5.1), and ACN (5.8) (extraction conditions: initial concentration of rutin = 0.5 mg·L^−1^, sample loading volume = 1000 *μ*L, aspirating/dispensing cycles = 15 cycles, sample pH = 6, and mass of sorbent = 2 mg). Figure 4(b) shows that propan-2-ol gave the highest desorption ability of rutin and was hence used as the eluting solvent in subsequent experiments. Dramou et al. [49] attributed that the ability of an eluent to desorb an analyte is due to the chemical interactions between the analyte and the sorbent. Rutin is a very polar compound due to the multiple OH groups and the attached disaccharide, and, as such, it could be expected that the more polar solvent (in this case MeOH) should have the highest desorption ability. According to a study by Mukherjee et al. [50], rutin and other flavonoids were detected in methanol, a polar solvent, when compared to other solvents such as n-hexane, chloroform, ethyl acetate, acetone, and even water. In the present study, MeOH was employed in the adsorption experiment, suggesting that certain analytes may have displayed a stronger affinity for the solvent than for the sorbent, leading to their elution with the solvent. Consequently, only a limited amount of the analyte remained on the sorbent bed, resulting in a minimal quantity being desorbed in the desorption studies.

### 3.4. Effect of Concentration of Standard Rutin

The effect of the concentration of rutin was also investigated to determine the concentration that aids the highest enrichment. The investigated concentrations were 0.5, 1, 1.5, and 2 mg·L^−1^ (extraction conditions: aspirating/dispensing cycles = 15 cycles, sample loading volume = 1000 *μ*L, elution solvent = propan-2-ol, sample pH = 6, and mass of sorbent = 2 mg). As depicted in Figure 4(c), when using a concentration of 2 mg·L^−1^, maximum amount of rutin was enriched. This then implied that a higher concentration of the standard analyte aids a higher enrichment of the analyte because there are more analyte ions to be adsorbed. According to a study by Rais et al. [51], when the analyte ions interact with the sorbent material, an escalation in their concentration leads to the saturation of binding sites. Initially, these binding sites are open for sorption, meaning that elevating the analyte concentration results in the occupation of more binding sites on the sorbent material. However, once all the binding sites are filled, the sorbent material reaches a state of saturation.

### 3.5. Effect of pH on the Sorbent

**Figure 5:**
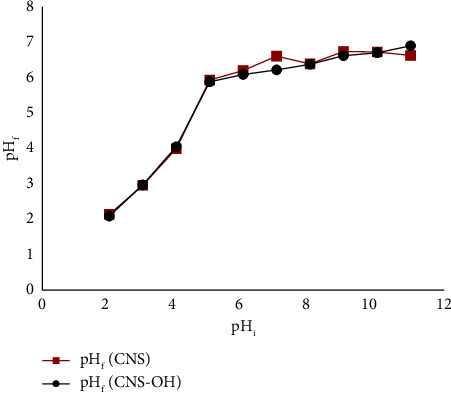
pH_i_ versus pH_f_ for the determination of point of zero charge of the HCNSs sorbent (*n* = 3).

The oxidation of HCNSs materials induced surface modifications, incorporating more oxygen-containing groups that attract the adsorption of the target analyte. The graph depicting initial pH (pH_i_) versus final pH (pH_f_) (Figure 5) was generated. The pHpzc is identified as the value at which pH_f_ remains nearly constant. In this experimental setup, the pHpzc was determined to be 6.5. Loading the rutin analyte onto the sorbent bed revealed increased adsorption in acidic conditions because at pH < pHpzc, the net charge on the surface of the HCNSs is positive [52]. However, there was only a slight difference in the adsorption capacity between acidic and basic media, with almost the same amount of rutin being adsorbed. At pH 7, less rutin was observed to be adsorbed onto the sorbent bed.  Figure 4(d) shows that the optimal pH for rutin extraction was determined to be pH 2 (extraction conditions: initial concentration of rutin = 2 mg·L^−1^, sample loading volume = 1000 *μ*L, elution solvent = propan-2-ol, aspirating/dispensing cycles = 15 cycles, and mass of sorbent = 2 mg), aligning with similar findings by Gholizadeh et al. [53]. Their study on MWCNTs showed maximum rutin adsorption at pH 2. The presence of numerous hydroxyl groups in rutin makes pH a crucial factor in the adsorption process. In high pH values, flavonoids dissociate into anions, causing surface functional groups to be either neutral or negatively charged, increasing electrostatic repulsion and subsequently decreasing adsorption capacity. Ran et al. [54] also noted that at pH ≤ 4, analytes exist in a neutral form, facilitating intermolecular interactions and yielding the highest enrichments. At pH > 4, flavonoid analytes exist in anionic form, resulting in less interaction with the sorbent. This observation stems from the pH-dependent ionization states of flavonoids, influencing the analytes' state in solution and, consequently, extraction efficiency. A study by Lazo-Cannato et al. [52] revealed that at acidic pH, phenol molecules are either in their acidic or neutral form. In their neutral form, the aromatic ring could have a greater electrophilic character toward the *π* electrons on the surface of the sorbent which increases the *π*–*π* interaction, thereby increasing the adsorption efficiency.

### 3.6. Effect of Sample Loading Volume

The quantity of standard rutin introduced into PT-*µ*SPE plays a crucial role in rutin enrichment, as the enrichment efficiency hinges on the loading sample solution volume. Therefore, the investigated sample loading volumes were 300, 500, and 1000 *μ*L (extraction conditions: initial concentration of rutin = 2 mg·L^−1^, aspirating/dispensing cycles = 15 cycles, elution solvent = propan-2-ol, sample pH = 2, and mass of sorbent = 2 mg). It was noted that loading PT-*µ*SPE with 500 *μ*L resulted in a higher rutin enrichment than the other volumes (Figure 4(e)). When the volume of the sample solution was increased to 1000 *μ*L, the amount of rutin adsorbed was observed to decrease because of shear pressure. A study by Bielicka-Daszkiewicz and Voelkel [55] highlights the effects of sample loading volume on the sorbent bed-analyte relationship. The study states that the sample volume that is loaded onto the sorbent bed can lead to a loss of analyte. This coincides with the observations in this study that a larger volume could displace the already adsorbed rutin during the aspirating/dispensing cycles. Consequently, 500 *μ*L was deemed the optimal value and was employed in subsequent experiments. Wang et al. [56] observed a parallel trend and attributed it to the partial loss of analytes from the sorbent bed associated with high sample volumes.

### 3.7. Effect of Mass of Sorbent

The sorbent's mass plays a role in rutin enrichment, prompting an investigation at 1, 1.5, 2, and 3 mg (extraction conditions: initial concentration of rutin = 2 mg L^−1^, sample loading volume = 500 *μ*L, elution solvent = propan-2-ol, sample pH = 2, and aspirating/dispensing cycles = 15 cycles). It was noted that at 1.5 mg, the enrichment of rutin was maximized, establishing it as the optimal mass (Figure 4(f)). Gomes et al. [57] concluded that an increase in sorbent mass provides more active sites for the removal of desired analytes. Consequently, 1.5 mg was employed in the analysis of real samples. Hu et al. [58] similarly observed a trend where extraction efficiency decreased with an increase in sorbent mass, attributing it to increased sorbent aggregation, reducing the accessible surface for sorbent–analyte interactions. This study witnessed a comparable trend.

## 4. Method Validation

The PT-*µ*SPE method was validated using rutin standards covering a linear range of 0.7–50 mg·L^−1^. Linearity, limit of detection (LOD), and limit of quantification (LOQ) are the analytical parameters that were evaluated. The correlation of determination equation *y* = 36,397*x* − 9442.2 from the calibration curve of the rutin standards gave a good linearity (*R*^2^=0.9991). The slope (*S*) and the intercept (*σ*) obtained from the correlation of determination were 36,397 and 9442.2, respectively. The LOD and LOQ were calculated from the standard deviation of the *y*-intercept and the slope, affording the equations LOD = 3.3 *σ*/*S* and LOQ = 10 *σ*/*S*, respectively. The LOD was calculated to be 0.604 mg·L^−1^, and the LOQ was calculated to be 1.830 mg·L^−1^. The results of the analysis of variance (ANOVA) test from the calibration curve are shown in Table 1. The *p* value was less than 0.05 which indicates that the linear fit is significant.

## 5. Real Sample Analysis

**Figure 6:**
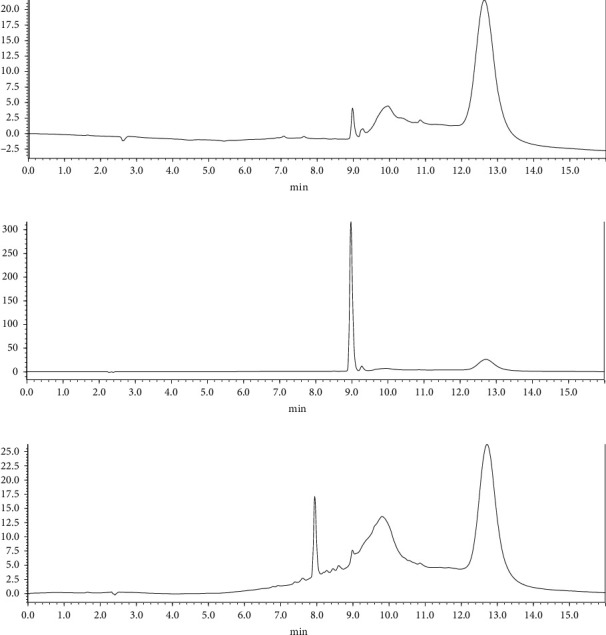
Chromatograms of (a) 0.7 mg·L^−1^ rutin standard, (b) 50 mg·L^−1^ rutin standard, and (c) *M. oleifera* sample.

The applicability and reliability of the PT-*µ*SPE method were investigated by applying it to a *M. oleifera* leaf extract sample contained rutin under the optimized conditions as described above. The investigation was done in triplicate measurements (*n* = 3).  Figure 6 shows the chromatograms of (a) a 0.7 mg·L^−1^ rutin standard, (b) 50 mg·L^−1^ rutin standard, and (c) *M. oleifera* leaf extract. Rutin was observed to elute at 8.9 min and had a maximum wavelength at 359 nm. Rutin was observed to have strong UV-vis absorption peaks at 255 and 360 nm by Deepika et al. [59]. The relative standard deviation (RSD) of the three sample runs was found to be 3.26%. Since RSD < 10%, the method is reliable and repeatable for the analysis of complex samples. A study by Du et al. [60] concluded the PT-*µ*SPE method to be simple, sensitive, and reproducible.

### 5.1. Comparison of the PT-µSPE Method With Other Methods

The method proposed in this study (PT-*µ*SPE) for the extraction of rutin from *M. oleifera* leaves was compared to other reported methods. As demonstrated in Table 2, the proposed method has a high sensitivity and a wide linear range and is reproducible. Other advantages of the proposed method are its simplicity, low solvent consumption, short extraction times, and no evaporation step. This method can also be used for the preconcentration of analytes in complex samples.

## 6. Conclusion

The successful extraction of rutin from *M. oleifera* leaves using PT-*µ*SPE with carbon nanospheres as the sorbent was demonstrated. This affirmed the effectiveness of activated HCNSs in rutin extraction. The oxidation of HCNSs material introduced more oxygen-containing groups, enhancing the adsorption of rutin on the carbonaceous material's surface. Rutin exhibited optimal adsorption at pH 2 with an initial concentration of 2 mg·L^−1^. Propan-2-ol emerged as the most effective eluting solvent for rutin desorption from the sorbent bed, with an optimal sorbent mass of 1.5 mg. Additionally, employing 15 aspirating/dispensing cycles and a loading volume of 500 *μ*L yielded the optimal extraction of rutin from *M. oleifera*. The achieved low LOD for rutin attested to the method's suitability for extracting rutin even at trace levels from complex samples. A calculated RSD of 3.26% indicated the reliability and repeatability of the PT-*µ*SPE method for analyzing complex samples.

## Figures and Tables

**Table 1:** Results of the analysis of variance (ANOVA) test for the calibration curve.

	***df***	**SS**	**MS**	***F***	**Significance *F***
Regression	1	2.32E + 12	2.32E + 12	3189	1226E − 05
Residual	3	2.18E + 09	7.26E + 08		
Total	4	2.32E + 12			

**Table 2:** Comparison of the proposed method with other reported methods.

**Method**	**Linear range**	**R** ^2^	**LOD**	**Precision (%RSD)**	**Ref.**
DES-UAE^a^	10–1000 *μ*g·g^−1^	0.9991	0.77 *μ*g·g^−1^	2.2	[61]
MGO/MHNTs@MIPs^b^	1–80 *μ*g·mL^−1^	0.9969	0.27 *μ*g·mL^−1^	Intraday (1.35) Interday (3.15)	[62]
ILMAE^c^	30–300 mg·L^−1^	0.99917	5.3 *μ*g·L^−1^	1.02–1.12	[63]
PT-*µ*SPE	0.7–50 mg·L^−1^	0.9991	0.604 mg·L^−1^	3.26	This work

^a^Deep eutectic solvent-based ultrasound-assisted extraction.

^b^Molecularly imprinted polymers on the surface of magnetic halloysite nanotubes with magnetic graphene oxide.

^c^Ionic liquid-based microwave-assisted extraction.

## Data Availability

Raw data are available upon request.
